# Migraine Headaches in a Pediatric Patient With SETD5-Related Neurodevelopmental Disorder and Inflammatory Comorbidities: A Case Report

**DOI:** 10.7759/cureus.112806

**Published:** 2026-07-16

**Authors:** Tareq M Hanna, David Elyas, Joy Lerner

**Affiliations:** 1 Pediatric Neurology, Henry Ford Hospital, Detroit, USA

**Keywords:** intellectual disability, migraine, neurodevelopmental disorder, pediatric headache, setd5

## Abstract

SETD5-related neurodevelopmental disorder is a rare genetic condition caused by a mutation in the SETD5 gene, which has previously been shown to cause intellectual disability (ID) along with dysmorphic facies, behavioral difficulties, congenital heart defects, and developmental delays. This report describes an eight-year-old patient with known SETD5 and inflammatory conditions who presented for evaluation of severe headaches consistent with migraines. This patient had associated photophobia and nausea, as well as pain around the eye that was severe enough to awaken him and was only relieved with rizatriptan. To our knowledge, migraine headaches have not previously been described in association with SETD5-related neurodevelopmental disorder. We hope that by sharing this patient’s presentation and medical history, this report will broaden the clinical spectrum of SETD5 and its implications.

## Introduction

Intellectual disability (ID) is defined as a cognitive deficit compared to peers and is most commonly noticed in childhood. Recently, the prevalence of ID in childhood was reported as ranging from 1.33% to 2.19% [1]. The SETD5 gene has been found to code for a highly conserved methyltransferase that exhibits high levels of expression in the brain during different developmental stages, helping promote central nervous system development. Loss-of-function mutations in this methyltransferase have been found to be associated with moderate to severe ID [2,3]. Along with ID and dysmorphic facial features, there have been various reported neurological complications associated with SETD5, including hypotonia (39.2%), gait abnormalities (35.7%), and seizures (14.2%) [4]. Given its role as a histone H3K4 methyltransferase regulating cortical gene transcription, SETD5 dysfunction may plausibly lower cortical excitability thresholds and predispose affected individuals to migraine, though this remains to be formally established. To our knowledge, no literature to date has described the presence of severe childhood migraine headaches in SETD5 patients. Here we report a pediatric patient with SETD5 who has comorbid debilitating migraine headaches.

## Case presentation

An eight-year-old Caucasian man with Juvenile Idiopathic Arthritis (JIA), Chronic Recurrent Multifocal Osteomyelitis (CRMO), attention-deficit/hyperactivity disorder (ADHD), a history of speech delay but average intellect, and a SETD5-related disorder (c.586C>T; p.Q196X) presented for the evaluation of severe headaches that had been present for three months. The headaches occur for 1-3 days per month. The patient’s mother stated he has had associated episodes of eye pain and photophobia. Rheumatology and ophthalmology had both evaluated the patient and did not find rheumatologic or structural ophthalmologic concerns, respectively, prompting evaluation by neurology.

The headaches characteristically occur in the early morning hours, often awakening the patient from sleep, and can last anywhere from a few hours to the entire day. It is important to note that these represent nocturnal awakening headaches rather than headaches present upon waking after a full night of sleep, a distinction relevant to the differential diagnosis of raised intracranial pressure (ICP). Patient identifies significant photophobia, which has been partially managed using sunglasses and hats. He has also had vomiting with some of these episodes. In one of these episodes, he woke up screaming in severe pain due to sensitivity to his nightlight and had multiple bouts of fecal incontinence and non-bloody, non-bilious emesis prior to falling back asleep with the onset of his prescribed Rizatriptan’s effect. His symptoms have worsened with each episode. Awakening with headaches prompts evaluation for increased ICP.

Review of systems did not show any other abnormalities of concern. Patient was alert and interactive with appropriate attention and behavior for age. The mental status examination was notable for intact orientation, appropriate comprehension, and ability to follow commands. Cranial nerve examination was normal, including intact pupillary responses, extraocular movements without evidence of nystagmus or ophthalmoplegia, symmetric facial movements, intact hearing, normal palate elevation, and midline tongue protrusion. Motor examination demonstrated normal bulk and tone with 5/5 strength throughout bilateral upper and lower extremities. No pronator drift was appreciated. Deep tendon reflexes were symmetric and within normal limits throughout. Sensory examination was intact to light touch throughout all extremities. Coordination was intact with normal finger-to-nose and heel-to-shin testing. Gait was normal with appropriate balance and coordination. No abnormal movements, tremor, or focal neurological deficits were identified.

Patient has a past medical history of coarctation of the aorta, ventricular septal defect, hypotonia, speech delay, JIA, and CRMO, for which he was being managed with a pediatric corticosteroid regimen at standard dosing. Blood pressure was normal for age. Fundoscopic examination revealed no papilledema, effectively excluding steroid-induced benign intracranial hypertension as a contributing diagnosis. Sinusitis was excluded clinically based on the absence of facial pain, nasal congestion, purulent discharge, and fever. Sleep deprivation was not an independent trigger in this patient, as disrupted sleep was a direct consequence of nocturnal headache awakening rather than a primary contributing factor. Excessive screen time was not formally assessed at the time of evaluation but warrants consideration in future follow-up visits. No significant psychosocial stressors were identified. The mother reports that she had a few migraines during pregnancy, but none since, and the father reports only having two migraines in his entire life. There is no other family history of headaches.

**Figure 1:**
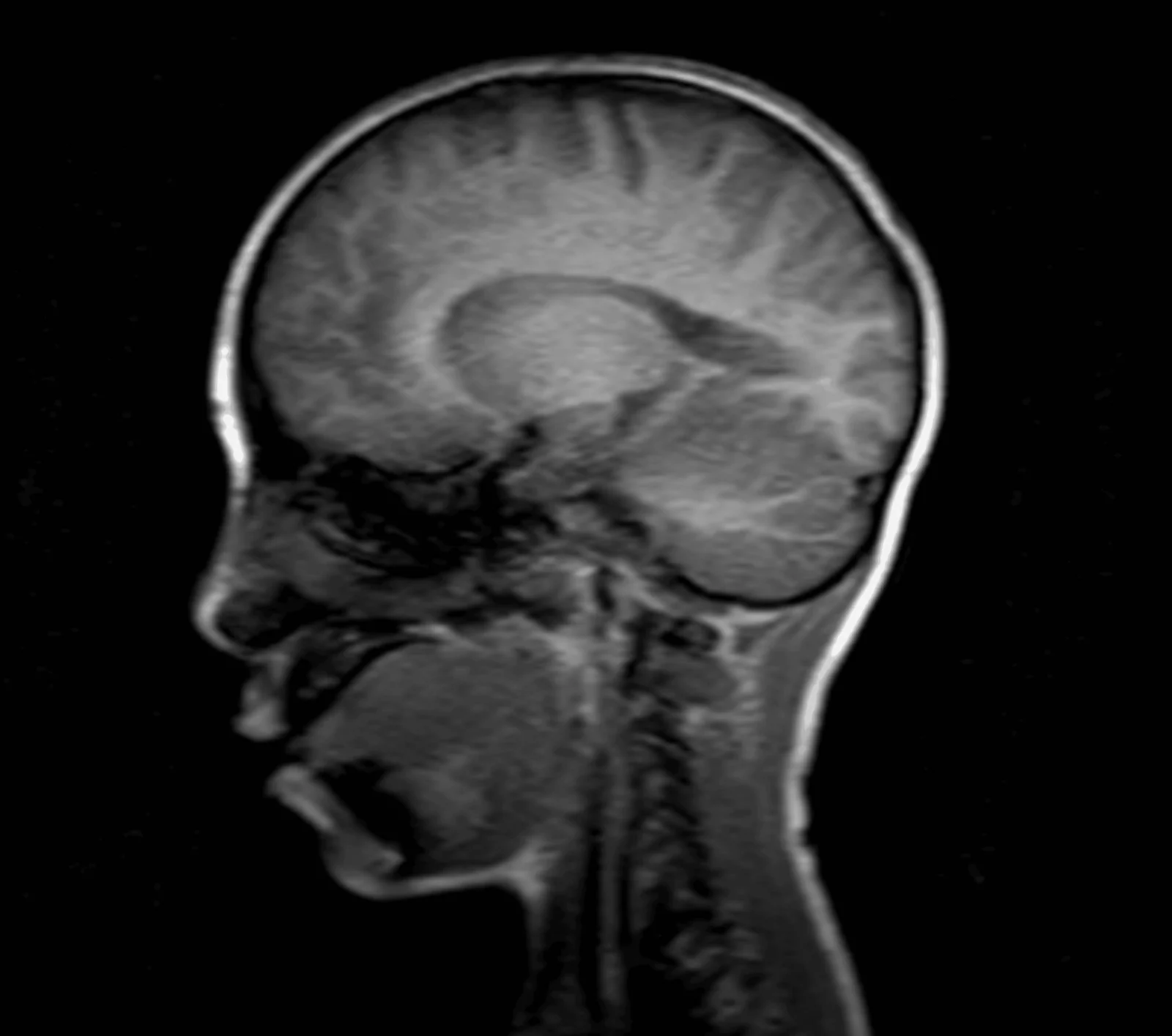
Mid-sagittal T1-weighted image. Normal cortical gyral pattern, midline structures, corpus callosum, brainstem, and cerebellar vermis. No tonsillar herniation or cervicomedullary junction abnormality.

**Figure 2:**
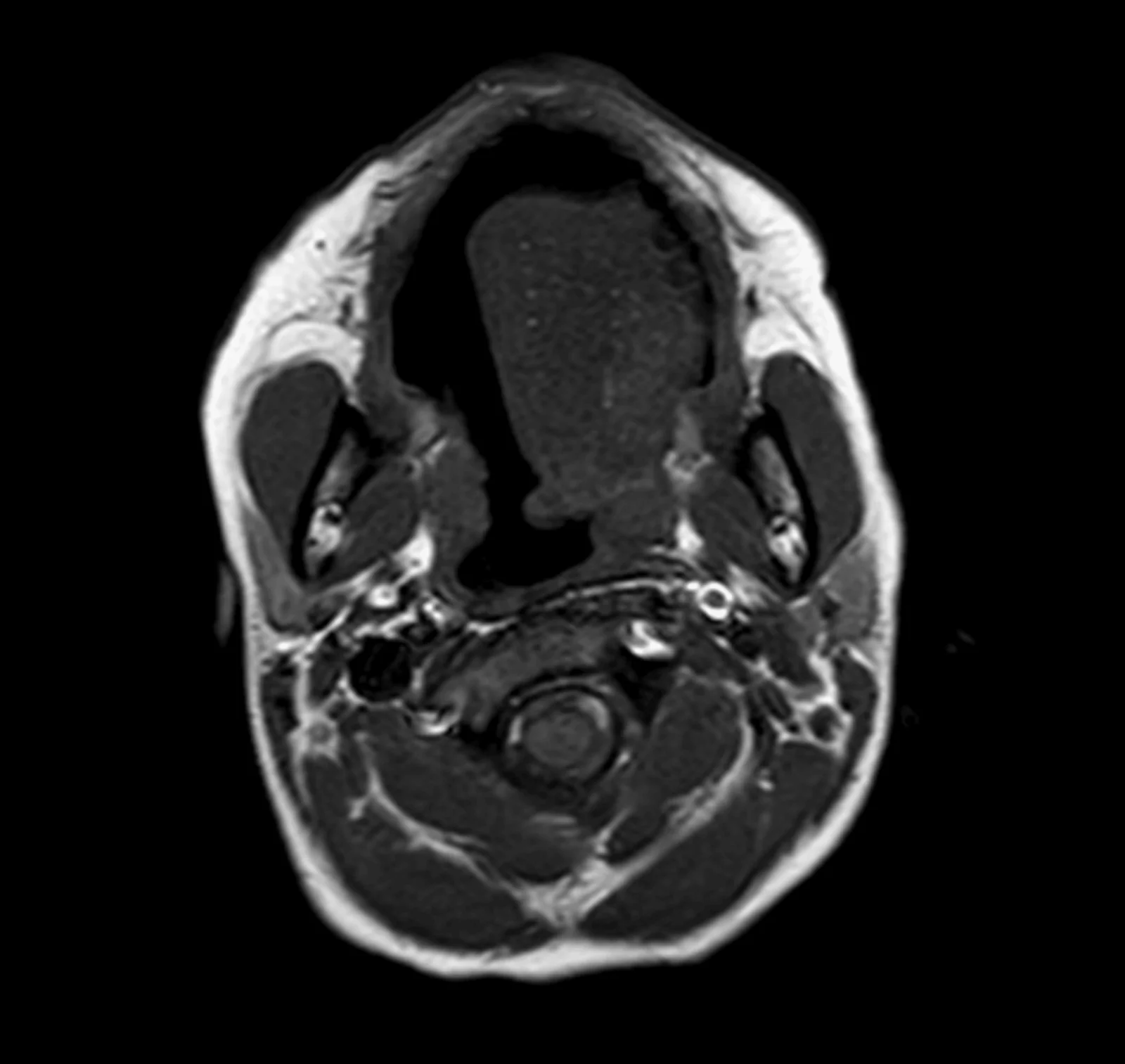
Axial T2-weighted image at the level of the posterior fossa. Normal signal intensity of the cerebellum and brainstem. Patent fourth ventricle and no evidence of obstructive hydrocephalus.

**Figure 3:**
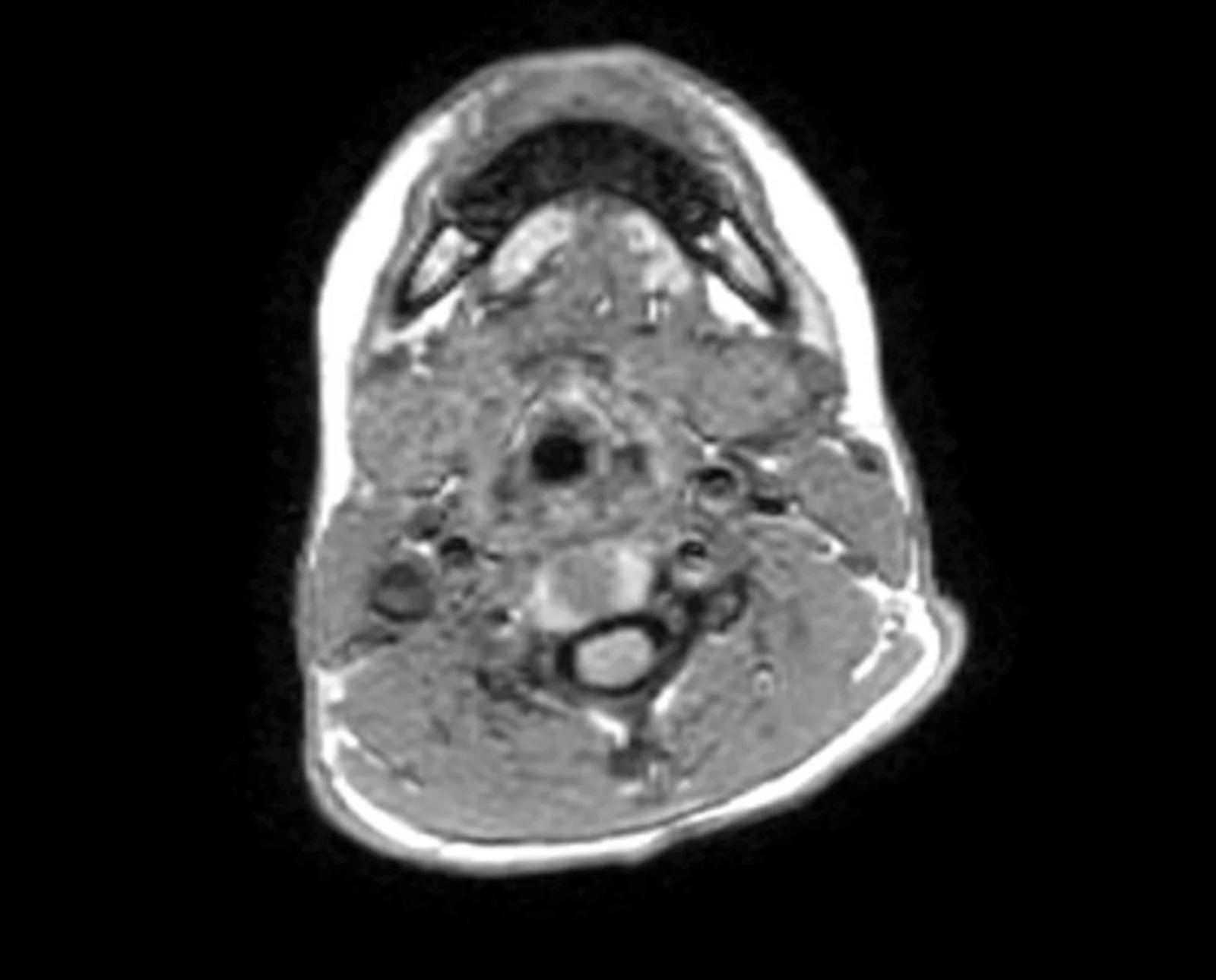
Axial T2-weighted image at the level of the upper cervical spine. Normal cervical cord signal without syrinx or cord compression. No craniocervical junction anomaly.

The diagnosis of migraine without aura was established using the International Classification of Headache Disorders, 3rd edition (ICHD-3) criteria adapted for pediatric patients. Per ICHD-3, migraine without aura in children requires: (A) at least five attacks fulfilling criteria B-D; (B) headache lasting 2-72 hours when untreated; (C) at least two of the following: unilateral or bilateral location, pulsating quality, moderate-to-severe intensity, or aggravation by routine physical activity; and (D) at least one of nausea/vomiting or photophobia/phonophobia during the headache. Our patient fulfilled all four criteria: he experienced more than five discrete episodes over three months, each lasting several hours to a full day; his pain was severe in intensity and worsened by light exposure and activity; and he had documented photophobia and vomiting with episodes. Magnetic resonance imaging (MRI) of the brain with and without contrast was performed under sedation and demonstrated normal findings (Figures 1-3). His Pediatric Migraine Disability Assessment (PedMIDAS) score was 6, corresponding to Grade I (little to no disability; score range 0-10) [5]. Daily prophylactic medication was not indicated given the infrequency of his migraines and his Grade I PedMIDAS score, as prophylaxis is generally reserved for patients with Grade II disability or higher [5]. The treatment plan is to continue Rizatriptan 5 mg orally as needed for acute attacks, with each episode lasting anywhere from a few hours to a full day before resolution with medication. EEG was not performed as the clinical presentation was not suggestive of a seizure disorder, with no witnessed convulsive episodes, post-ictal periods, or epileptiform features.

## Discussion

Despite there being various types of ID, they can often be characterized based on specific features such as language and motor deficits, physical malformations, and behavioral problems. The underlying genetic cause of ID is what determines which characteristics are present. Previous studies found that the SETD5 gene is located on chromosome 3p25.3 and codes for SET domain-containing protein 5 [6-8]. The protein belongs to the SET methyltransferase family and notably catalyzes methylation of histone H3 and H4 lysine residues, thus regulating gene transcription [6,8].

Previous studies have established that SETD5 mutations can lead to variable ID, autism, cardiac defects, gastrointestinal defects, renal defects, skeletal abnormalities, and abnormal facies [8,9]. However, to our knowledge, there has not been any mention in the current literature of migraines as part of the SETD5 presentation [3-4,6,8]. The patient’s treatment plan is to continue Rizatriptan, as an MRI of the brain revealed no evidence of a structural cause for his red flag symptoms of early morning headaches and vomiting. To our knowledge, no prior cases of migraine in SETD5 patients have been reported in the literature, making direct comparison with existing cases impossible at this time. The specific mutation identified in our patient (c.586C>T; p.Q196X) results in a premature stop codon and presumed loss of function, consistent with the broader SETD5 loss-of-function phenotype described in the literature [6-8]. It is also worth noting that this patient carries a diagnosis of ADHD, which has itself been associated with headaches in the pediatric population; however, given the severity, frequency, and clear migrainous features of his headaches, ADHD alone is unlikely to account for the clinical presentation. Additionally, his corticosteroid use for JIA and CRMO was considered a potential contributor, as steroid use is a recognized cause of benign intracranial hypertension; however, this was excluded by fundoscopic examination revealing no papilledema.

SETD5 has been observed to be highly expressed in the cerebral cortex, the intestine, and the eye [6]. Based on the brain MRI, there were no structural abnormalities that could explain these headaches or any other structural irregularities of the brain.

Early-morning headache with vomiting is a well-recognized red flag pattern that raises concern for raised ICP, and our clinical team appropriately pursued brain MRI to exclude structural pathology. The principal differential diagnoses considered included idiopathic intracranial hypertension (pseudotumor cerebri), posterior fossa mass or other space-occupying lesion, hydrocephalus, and Chiari malformation type I, all of which can present with morning headaches and vomiting due to positional or nocturnal ICP fluctuations. Normal MRI with and without contrast, including no evidence of papilledema, tonsillar herniation, ventricular enlargement, or parenchymal lesion, effectively excluded these structural causes. Cyclic vomiting syndrome and abdominal migraine, recognized as ICHD-3 episodic syndromes associated with migraine, were also considered but were not the primary diagnosis given the predominance of cephalic pain. The early-morning timing of this patient’s attacks most likely reflects the known phenomenon of sleep-related migraine, in which the end of the overnight rapid-eye-movement (REM) sleep cycle is associated with autonomic changes and serotonergic fluctuations that can trigger attacks in susceptible individuals.

Although the precise mechanism by which SETD5 dysfunction might contribute to migraine susceptibility remains speculative, several lines of evidence support a plausible biological link. SETD5 encodes a histone H3K4 methyltransferase that regulates transcription of genes critical for cortical neuronal development and function [6-8]. Loss-of-function mutations in SETD5 are associated with aberrant gene expression in excitatory cortical neurons, and growing evidence implicates epigenetic dysregulation of histone methylation in the pathophysiology of migraine [10,11]. Altered histone methylation can disrupt the transcriptional balance between excitatory and inhibitory neurotransmission, potentially lowering the cortical spreading depression (CSD) threshold, the electrophysiological correlate of migraine aura and a key driver of trigeminovascular activation [10]. Furthermore, SETD5 is highly expressed in the cerebral cortex during key developmental windows [6], and disruption of its activity may result in lasting changes in cortical excitability that predispose affected individuals to migraine. The epigenetic regulation of calcitonin gene-related peptide (CGRP), a neuropeptide central to migraine pathogenesis, has also been shown to be sensitive to histone modification [11], providing an additional mechanistic link between SETD5 dysfunction and migraine susceptibility.

The contribution of this patient’s inflammatory comorbidities, JIA and CRMO, to his migraine presentation also warrants consideration. Both conditions are characterized by chronic systemic inflammation driven by pro-inflammatory cytokines, including TNF-α, IL-1β, and IL-6 [12]. Elevated levels of these cytokines have been documented in pediatric migraine patients and are thought to sensitize the trigeminovascular system, lower nociceptive thresholds, and contribute to the neuroinflammatory cascade underlying migraine attacks [12,13]. It is therefore plausible that the chronic inflammatory milieu associated with JIA and CRMO may have lowered this patient’s threshold for migraine, either independently of SETD5 or synergistically with it. Disentangling the relative contributions of the genetic (SETD5) and inflammatory factors to his headache phenotype is not possible from a single case, and prospective studies examining migraine prevalence in SETD5 cohorts as well as in children with systemic inflammatory diseases will be necessary to clarify these relationships.

## Conclusions

Our patient presented with migraine headaches and normal neuroimaging, suggesting a potential expansion of the neurological phenotype associated with SETD5-related neurodevelopmental disorder. Despite its wide spectrum of phenotypes, to our knowledge, there have been no previous reports in the literature of migraine headaches being associated with this condition. We share this case in the hopes that it will add to our growing knowledge of SETD5 and motivate further research into the potential comorbidities and presentations of this disease.
